# Extracellular matrix signatures of human primary metastatic colon cancers and their metastases to liver

**DOI:** 10.1186/1471-2407-14-518

**Published:** 2014-07-18

**Authors:** Alexandra Naba, Karl R Clauser, Charles A Whittaker, Steven A Carr, Kenneth K Tanabe, Richard O Hynes

**Affiliations:** 1 David H. Koch Institute for Integrative Cancer Research, Massachusetts Institute of Technology, Cambridge, MA 02139, USA; 2 Howard Hughes Medical Institute, Massachusetts Institute of Technology, 02139 Cambridge, MA, USA; 3 Proteomics Platform, Broad Institute of MIT and Harvard, 02142 Cambridge, MA, USA; 4 David H. Koch Institute for Integrative Cancer Research - Barbara K. Ostrom Bioinformatics and Computing facility at the Swanson Biotechnology Center, 02139 Cambridge, MA, USA; 5 Division of Surgical Oncology, Massachusetts General Hospital Cancer Center, Boston 02114, MA, USA

**Keywords:** Extracellular matrix, Proteomics, Colorectal cancer, Metastasis, Tumor microenvironment, Matrisome

## Abstract

**Background:**

Colorectal cancer is the third most frequently diagnosed cancer and the third cause of cancer deaths in the United States. Despite the fact that tumor cell-intrinsic mechanisms controlling colorectal carcinogenesis have been identified, novel prognostic and diagnostic tools as well as novel therapeutic strategies are still needed to monitor and target colon cancer progression. We and others have previously shown, using mouse models, that the extracellular matrix (ECM), a major component of the tumor microenvironment, is an important contributor to tumor progression. In order to identify candidate biomarkers, we sought to define ECM signatures of metastatic colorectal cancers and their metastases to the liver.

**Methods:**

We have used enrichment of extracellular matrix (ECM) from human patient samples and proteomics to define the ECM composition of primary colon carcinomas and their metastases to liver in comparison with normal colon and liver samples.

**Results:**

We show that robust signatures of ECM proteins characteristic of each tissue, normal and malignant, can be defined using relatively small samples from small numbers of patients. Comparisons with gene expression data from larger cohorts of patients confirm the association of subsets of the proteins identified by proteomic analysis with tumor progression and metastasis.

**Conclusions:**

The ECM protein signatures of metastatic primary colon carcinomas and metastases to liver defined in this study, offer promise for development of diagnostic and prognostic signatures of metastatic potential of colon tumors. The ECM proteins defined here represent candidate serological or tissue biomarkers and potential targets for imaging of occult metastases and residual or recurrent tumors and conceivably for therapies. Furthermore, the methods described here can be applied to other tumor types and can be used to investigate other questions such as the role of ECM in resistance to therapy.

## Background

With more than 140,000 new cases diagnosed in 2012, colorectal cancer is the third most commonly diagnosed cancer type in both men and women in the United States. Thanks to prevention and, particularly, early detection, there has been a steady decrease in the number of deaths due to colorectal cancer over the last two decades. And yet, in 2012 it was estimated that colorectal cancer would claim the lives of 50,000 patients. Several genes have been directly implicated in the etiology of colorectal cancer and, despite the fact that tumor-intrinsic molecular mechanisms controlling colorectal carcinogenesis have been identified [1,2], novel prognostic and diagnostic tools as well as novel therapeutic strategies are still needed to prevent colon cancer progression.

Proteomics has become a method of choice to identify cancer-related biomarkers [3]. Within the last five years, over 35 studies published in peer-reviewed journal applied global proteomics techniques to the study of colorectal samples from patients (reviewed in [4,5]). These studies revealed a certain number of proteins (including extracellular matrix proteins, see Results and discussion section) up or down-regulated in cancer samples as compared with normal samples, which represent potential biomarkers. However, as discussed in the review by De Wit and colleagues, these studies have not yet been successfully translated to the clinic [4].

The extracellular matrix (ECM) is a complex meshwork of cross-linked proteins providing architectural support for cells. In addition, ECM proteins bind and present growth factors to cells, thus providing both biophysical and biochemical cues that are major regulators of cellular behavior [6,7]. The ECM is a major component of the tumor microenvironment and exerts many roles during tumor progression: it supports proliferation and survival of tumor cells; it contributes to the formation of the cancer stem cell niche and thus sustains primary tumor growth; it participates by its nature and/or architecture in the formation of a pro-invasive environment; and, finally, it contributes to the invasion of distant sites by participating in the formation of a microenvironment that will support tumor cell seeding and growth [8-10]. Classical pathology has used excessive ECM deposition (desmoplasia) as a marker of tumors with poor prognosis long before the composition and the complexity of the ECM was even uncovered. Recent studies have also suggested that the ECM can act as a barrier to drug delivery and can confer chemo-resistance to tumors [11,12]. The ECM thus appears of great interest for discovery of ways to predict, diagnose and cure cancer.

In order to characterize the ECM composition of tumors, we have developed a proteomics-based approach and have shown, using mouse models, that we can identify 100–150 ECM proteins in any given tissue or tumor sample [13]. Using human melanoma and mammary carcinoma xenograft models, we have demonstrated that tumors of different metastatic ability differ in both tumor- and stroma-derived ECM components [13,14]. Moreover, we showed that several tumor-derived ECM proteins, characteristic of highly metastatic tumors, play important causal roles in metastatic dissemination [14].

Having developed these systematic methods, we now wished to analyze the composition of the ECM of human patient samples. We report here the characterization of the ECM composition of metastatic colorectal cancer samples (both primary tumors and metastases to liver) and paired normal tissues (normal colon and liver tissue). We have been able to identify consistent changes in the ECM of i) colon tumors as compared with normal colon ECM; ii) primary tumors as compared with metastases derived from them. Based on these changes, we derived ECM protein signatures of primary colon carcinoma and primary colon tumor metastasis to liver. Comparisons of these signatures with available clinical gene expression array data show that subsets of these signatures correlate well with tumor progression and metastasis. We believe that these data sets will lead to the identification of more precise predictive signatures and to the development of assays, in particular serological measurements or immunohistochemical assays, which could be used by pathologists to improve cancer patient management and care.

## Methods

### Patient samples

For each of the three patients included in this study, we obtained a set of three samples: normal colon, colon tumor and its metastasis to liver. None of the patients had received chemotherapy prior to surgery and sample collection and were all diagnosed with stage IV metastatic colon cancer. When available, we obtained duplicate samples of some tissues. Samples were between 25 mg and 85 mg. To obtain enough material to characterize the composition of the normal liver ECM, we generated two pools of samples from 4 and 5 healthy patients respectively (reaching a total of approximately 450 mg per pool). Informed consent was obtained from all of the patients and none of the specimens came from minors. The anonymized specimens were obtained from the MGH tissue bank and were removed for medical reasons unrelated to this project. The specimens were analyzed in accordance with a protocol approved by the Massachusetts General Hospital’s Institutional Review Board (IRB).

### Tissue preparation and ECM protein enrichment

The tissue and tumor samples were homogenized with a Bullet Blender (Next Advance, Averill Park, NY) according to the manufacturer’s instructions. Sequential extractions of frozen samples of tumors were performed using the CNMCS compartmental protein extraction kit (Millipore, Billerica, MA) as previously described [13]. In brief, frozen samples were homogenized and extracted sequentially to remove preferentially (1) cytosolic proteins, (2) nuclear proteins, (3) membrane proteins, (4) cytoskeletal proteins leaving a final insoluble fraction enriched for ECM proteins, although different tissues behave somewhat differently in the ease of extraction so that proteins sometimes appear in more than one fraction. The effectiveness of extraction of specific proteins was monitored by immunoblotting using the following antibodies: rabbit anti-collagen I, mouse anti-GAPDH and rabbit anti-histones (Millipore, Billerica, MA), the rabbit anti-actin antibody (serum 14–1) was generated in our laboratory.

### Protein digestion, peptide fractionation, and mass spectrometry

The ECM-enriched samples were solubilized in urea, reduced, digested with PNGaseF, Lys-C, and trypsin as previously described [13]. The resulting peptides (~50 μg) were separated into 11 fractions by off-gel electrophoresis (OGE) according to isoelectric point over a pH range of 3–10 [13]. Each OGE fraction was analyzed by LC-MS/MS with an LTQ Orbitrap XL mass spectrometer (Thermo Fisher Scientific, San Jose, CA). Mass spectra were interpreted with Spectrum Mill. MS/MS spectra were searched against a UniProt database containing human (78,369 entries) sequences downloaded from the UniProt web site on June 30, 2010 with a set of common laboratory contaminant proteins (73 entries) appended. Peptides identified with a false discovery rate < 1.6% were assembled into identified proteins, and annotated as being ECM-derived or not as previously described [13]. Detailed information is provided as Additional file 1: Supplementary Methods. The raw mass spectrometry data have been deposited in the public proteomics repository MassIVE (http://massive.ucsd.edu) using the identifier: MSV000078555. The data should be accessible at ftp://MSV000078555:a@massive.ucsd.edu.

### Gene Set enrichment analysis

Gene Set Enrichment Analysis was performed using GSEA v2.0.12 (http://www.broadinstitute.org/gsea). We identified four clinical gene expression datasets which reported measurements on both primary colon tumors and metastases to liver. The sample type was used to define phenotypic classes for comparison. Probe sets were collapsed to unique gene symbols. Gene sets corresponding to the proteins in our ECM signatures of primary metastatic colon tumors (37 genes) or metastases to liver (7 genes) were created and the distributions of the genes for each of these signatures against the rank-ordered metastasis vs. primary colon tumor comparisons were characterized using GSEA with the default settings. A positive normalized enrichment score indicates enrichment in metastasis samples. A negative normalized enrichment score indicates enrichment in primary colon tumor samples. Each gene in the proteomics-derived gene set is ordered by its position in the ranked list of genes from the dataset and is assigned a rank metric score reflecting its position and the number of probes in the expression dataset. The leading-edge subsets of genes are those genes that appear in the ranked list before (for positive enrichment scores) or after (for negative enrichment scores) the point at which the running sum reaches its maximum deviation from zero. The leading-edge subset can thus be interpreted as the core that accounts for the gene set’s enrichment signal. Note that the analysis includes only those genes in the gene set that are also in the expression dataset. The raw GSEA data may be downloaded from: http://rowley.mit.edu/Hynes/Naba_GSEA_ColonCancer/.

## Results and discussion

### Proteomic analyses of ECM from normal tissues and tumors from colorectal patient samples

**Figure 1:**
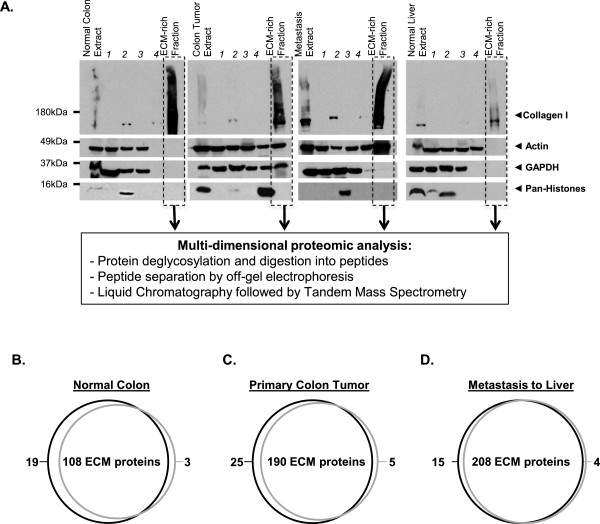
**ECM protein enrichment from tissues and tumors and reproducibility of the proteomic analysis. A**. The ECM protein enrichment and sequential extraction of intracellular components (steps 1 to 4) were monitored in each sample (normal colon, colon tumor, metastasis to liver and normal liver) by immunoblotting for collagen I (ECM marker), actin (cytoskeletal marker), GAPDH (cytosolic marker), and histones (nuclear marker). The insoluble fraction remaining after serial extraction was enriched for ECM proteins and largely depleted for intracellular components. **B-D**. Intra-patient reproducibility was assessed by comparing the ECM compositions of two distinct pieces of the same normal colon **(B)**, primary colon tumor **(C)** or metastasis to liver **(D)** from patient 1. Venn diagrams show the intra-patient reproducibility in terms of matrisome proteins.

We obtained from Massachusetts General Hospital’s tissue bank patient-matched metastatic colorectal cancer samples (primary tumor and paired metastases to liver) and normal colonic tissue from three patients. We also obtained normal liver tissue from healthy donors (see Methods). ECM proteins were enriched from normal tissues or tumors using the subcellular fractionation protocol previously described [13]. Figure 1A shows the efficiency of the sequential extraction protocol leading to significant enrichment of collagen I (other ECM proteins were similarly retained in the final insoluble fraction; data not shown) and concomitant depletion of intracellular proteins (actin, GAPDH, histones) in the final ECM-enriched samples.

The composition of the ECM-enriched fractions obtained was subsequently characterized by mass spectrometry. The complete proteomic data sets and the matrisome subsets (ECM and ECM-associated proteins) are presented in Additional files 2 and 3.

### Evaluation of intra-patient reproducibility

To evaluate the reproducibility of our approach, we conducted analyses on two distinct pieces from each tissue or tumor from patient 1 (Figure 1B-D). We observed good overlap between the ECM proteins detected in each of the duplicate samples analyzed. This was true not only for the duplicate normal colon samples analyzed (Figure 1B) but also for the duplicate colon tumor samples (Figure 1C) and the duplicate metastasis samples (Figure 1D). We observed similar reproducibility for normal colon and colon tumor samples from patient 2 (Additional file 4). Of note, we had observed similar results from normal murine lung and colon tissues [13]. These results argue against significant intra-tumoral heterogeneity detectable at this level of analysis; it appears that the sample size (25-85 mg of tissue) was sufficient to average out any spatial heterogeneity in the ECM. It is also worth noting that we detected more ECM proteins in the tumor samples (primary colon tumor and metastasis to liver) than in the normal colon samples, which may reflect the desmoplasia that often accompanies tumor progression.

### Comparison of the ECM composition of tissues and tumors across different patients

**Figure 2:**
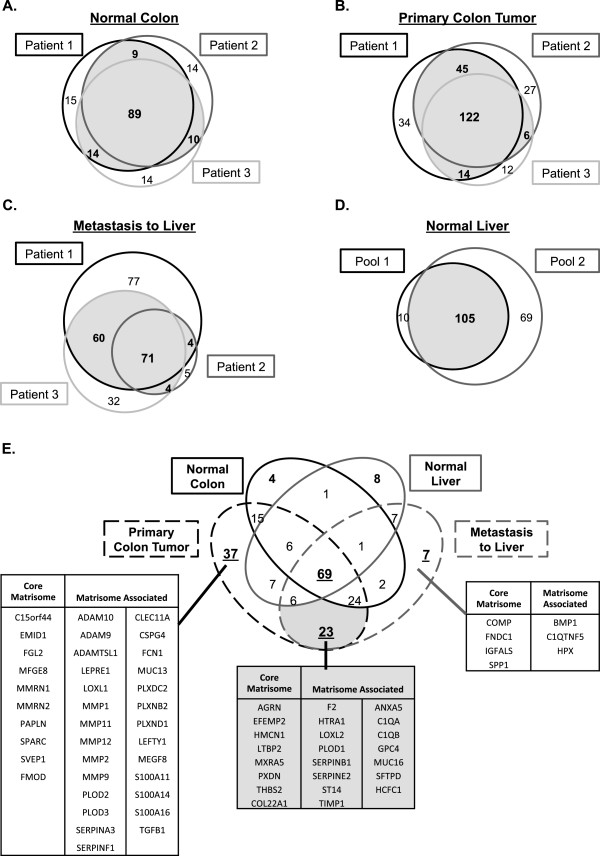
**Definition and comparison of the matrisomes of the metastatic colon cancer and control tissue. A-C**. Venn diagrams show the numbers of ECM proteins overlapping among the ECM-enriched fractions of the three patients’ normal colons **(A)**, primary colon tumors **(B)** and metastases to liver **(C)**, respectively. For patients for whom we analyzed duplicate samples of the same tissue or tumor, we chose the sample with the most abundant ECM protein content (see Additional file 2). We define the matrisome of a given tissue as the ensemble of proteins detected in at least two out of the three patients (grey). **D**. Venn diagram shows the number of proteins overlapping between the ECM of two pools composed of 4 and 5 normal liver samples respectively. The normal liver matrisome is composed of 105 proteins (grey). **E**. Venn diagram shows comparisons among the metastatic colon cancer matrisomes (primary colon tumor and associated metastases to liver) and control tissue matrisomes (normal colon and normal liver). ECM signatures of primary metastatic colon tumors and associated metastases are listed (see also Additional file 2C).

We next wanted to compare the ECM composition of samples (normal tissues or tumors) from different patients. Therefore, we extended our analyses to samples from two additional patients and compared the compositions of the ECM of normal colon (Figure 2A), primary colorectal tumor (Figure 2B), and metastases to liver (Figure 2C) with the data obtained for patient 1. When we compared the ECM composition of the normal colon samples from three patients, we identified a set of 89 ECM proteins present in all three samples (Figure 2A). In addition we identified subsets of proteins present in two out of three samples (representing 10% to 12% of the proteins) and, finally, about 12% of the ECM proteins detected were patient-specific. The comparison of three primary metastatic colorectal tumor samples from three patients revealed that, again, the majority of proteins detected were found in all three samples (122 proteins; Figure 2B). We also identified subsets of proteins present in two out of three samples and finally, depending on the sample, 8% to 15% of the ECM proteins detected were patient-specific. The inter-patient variability was greater for the metastasis samples (Figure 2C). Although we detected a set of 71 proteins common to all three metastasis samples analyzed, the number of ECM proteins detected in the metastasis from patient 1 was twice the number detected in patient 2 and 1.5 times the number of proteins detected in patient 3. For each of the three tissue types analyzed, we observed a striking overlap, although it is worth noting that the inter-patient overlap is notably greater for normal colon samples than it is for colon tumor and metastasis samples, which may reflect the heterogeneity among tumor samples (primary tumors or metastases) as compared with normal tissues (see Conclusions).Because of the very small amount of extracellular matrix in the normal liver, we were not able to analyze reliably the liver ECM from individual patient samples (on average 50 – 75 mg). Instead, and because of the high inter-patient overlap observed with normal colon samples, we generated two pools of normal liver samples from healthy donors (pools were composed of 4 and 5 liver samples, respectively, and were approximately 450 mg each). ECM proteins could successfully be enriched from these pooled liver samples (Figure 1A). Moreover, this strategy allowed us to obtain enough ECM material to be analyzed by mass spectrometry. We identified 115 and 174 ECM proteins in each pool, and 105 of them were detected in both pools (Figure 2D).

**Figure 3:**
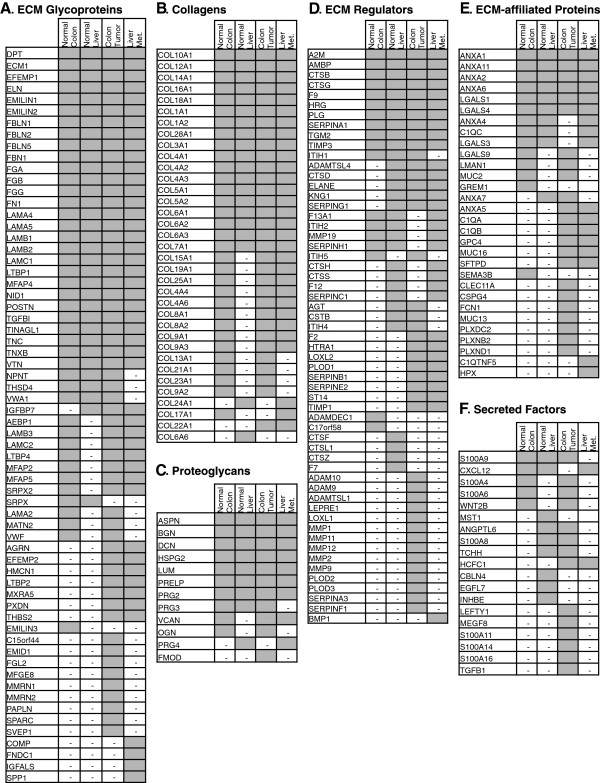
**Comparison of the metastatic colon cancer matrisomes and control tissue matrisomes.** Proteins included in this table were detected in at least two of the three patients for normal colon, colon tumor and metastasis samples, or in both pools of normal liver samples (see grey areas in Figure 2A-D). The proteins are subdivided into categories constituting the matrisome as follows: the core matrisome includes structural proteins such as ECM glycoproteins **(A)**, collagens **(B)** and proteoglycans **(C)**. In addition we defined three categories of ECM-associated proteins that are present at lower molar ratios: ECM regulators include ECM remodeling enzymes (crosslinking enzymes, proteases and their regulators; **D)**, ECM-affiliated proteins **(E)** and ECM-associated secreted factors (including growth factors, cytokines, etc.; **F)** Dash (-) indicates that the protein was not designated part of the matrisome of a given tissue (although in some instances, the protein was detected in one patient only, see Additional file 2B,C and Conclusions).

After evaluating the similarity among samples obtained from different patients, we wanted to compare the ECM compositions of normal tissues with those of primary and secondary tumors. We therefore defined, for each tissue or tumor type, its “matrisome” as the ensemble of proteins detected in at least two of the three patients studied. According to this definition, the matrisome of normal human colon comprises 122 proteins, the matrisome of primary colon tumors 187 proteins and the matrisome of metastases 135 proteins (grey areas in Figure 2A-C). To define the normal liver matrisome, we took the intersection (105 proteins) of the two pools analyzed (Figure 2D). We further subdivided each matrisome list into the protein categories we previously defined [13,15]: ECM glycoproteins, collagens and proteoglycans for core matrisome proteins and ECM-affiliated proteins, ECM regulators and secreted factors for ECM-associated proteins (Figure 3) [13,15].

### Definition of signatures of metastatic colorectal cancer and associated metastasis to liver

The comparison of the matrisome compositions of normal tissues (colon and liver) and colorectal tumors (primary tumors and metastases) revealed that a large fraction of proteins (69) are ubiquitously expressed and detected in all four tissue types (Figure 2E, Figure 3). We observed that half of the glycoproteins detected (Figure 3A) as well as most of the collagens (Figure 3B) and proteoglycans (Figure 3C) are common to the four tissue types. Components associated with the extracellular matrix, such as ECM regulators (that include ECM remodeling enzymes) or ECM-affiliated proteins and ECM-associated secreted factors (growth factors, cytokines etc.) are present at lower abundance in the ECM-rich samples (Additional file 2B) and are, for the most part, restricted to certain tissues (Figure 3, Additional file 2B). This comparison also revealed that the ECM composition of metastases to liver resembles more the ECM of primary colorectal tumors than that of normal liver. Importantly, we identified subsets of tumor-specific proteins: 37 proteins were characteristic of the colon tumor matrisome, 7 proteins were characteristic of the metastasis matrisome and 23 proteins were characteristic of both primary tumors and metastases (Figure 2E, Figure 3, and Additional file 2C).

### Gene Set enrichment analyses identify subsets of ECM-encoding genes strongly correlated with primary colon tumors or their metastases to liver

**Figure 4:**
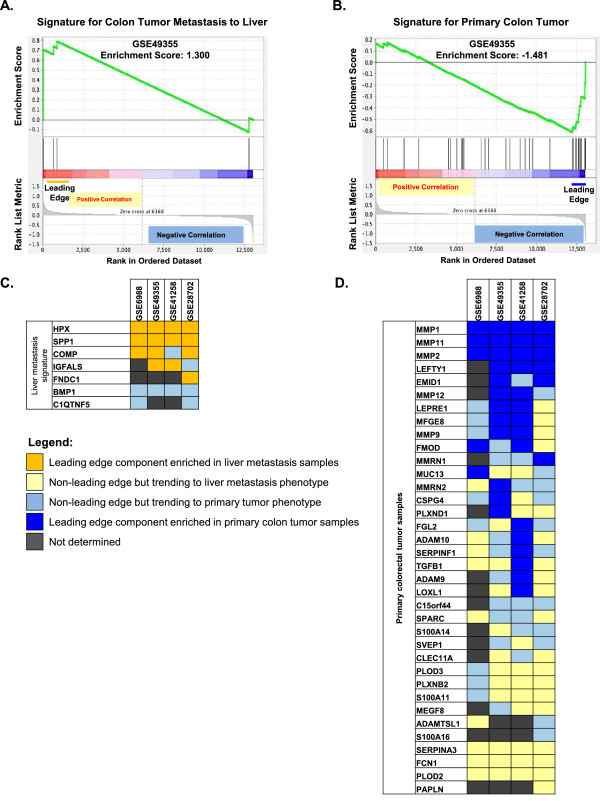
**Gene Set Enrichment Analysis.** Representative enrichment plot resulting from the comparison of the ECM proteomic signature of metastasis **(A)** or the ECM primary metastatic colon tumor signature **(B)** with the gene expression data set GSE49355 comprising 20 primary colon adenocarcinoma samples and 19 paired liver metastasis samples. The position of the leading edge is highlighted and the direction of the correlation indicated. The positions of the ECM signature genes along the class comparison for each gene set are indicated by vertical lines. **C - D.** Heat map shows enrichment of the ECM metastasis signature in metastasis microarray samples **(C)** and of the ECM primary colon cancer signature in primary colon cancer microarray samples **(D)**. Cells are colored according to the role of those genes in the enrichment based on their rank metric scores (Additional file 5B). Dark yellow cells indicate genes present in the leading edge of metastasis enrichment; dark blue indicates genes present in the leading edge of colon cancer enrichment; light blue indicates genes trending toward the colon cancer phenotype but not part of the leading edge; light yellow indicates genes trending toward the liver metastasis phenotype but not part of the leading edge. Grey cells indicate genes for which the expression level was not determined.

We next sought to explore potential correlations between our data and other clinical data sets. Accordingly, we used Gene Set Enrichment Analysis [16] to evaluate the relationship between the proteomics-derived ECM signatures for i) colon primary tumors and ii) metastases (Figure 2E, Figure 3; see Methods for further details) and microarray-based gene expression studies involving large cohorts of patients. The four relevant clinical gene expression data sets analyzed [17-20] represent a total of 289 primary colon tumor samples and 120 metastasis samples (Additional file 5). Comparisons were set up between colon tumor metastases to liver and primary tumor samples; hence a positive enrichment score will indicate enrichment in metastasis samples and a negative enrichment score, enrichment in primary colon tumor samples (Figure 4A,B and Additional file 5). The distributions of genes in our two signatures within these comparisons were characterized with GSEA and we consistently observed enrichment of our proteomics-based signatures in their corresponding phenotypic classes in the gene expression experiments. The metastasis signature was consistently enriched in the metastasis samples in all four comparisons, with enrichment scores ranging between 1.112 and 1.815 (Figure 4A, Additional file 6, left panels). The colon cancer signature set was enriched in the colon cancer class in all four comparisons, with enrichment scores ranging from -0.876 and -1.481 (Figure 4B, Additional file 6, right panels).

Next, we evaluated which of the genes from our ECM signatures consistently ranked most highly with regard to class association by comparing the composition of the various leading edge sets (Figure 4C,D, see Methods). Despite the fact that it is well known that protein levels do not necessarily correlate with mRNA levels and one would not expect one-to-one concordance, we identified a subset of three out of the seven genes from the metastasis signature (HPX, SPP1 and COMP) that were closely associated with the metastasis class in three out of the four clinical expression datasets (Figure 4C). We also identified a subset of four genes from the primary colon tumor signature (MMP1, MMP2, MMP11, and LEFTY1) that was associated with the colon cancer class in at least three out of the four clinical expression datasets tested (Figure 4D). An additional six genes (EMID1, MMP12, LEPRE1, MFGE8, MMP9 and FMOD) were strongly associated with primary colon cancers in two out of four clinical gene expression data sets. A recent gene expression profiling study by Lin and colleagues also identified osteopontin (SPP1, Secreted Phospho-Protein1) as being up-regulated in colorectal liver metastases as compared to primary tumors and normal liver, in accordance with our data [21]. The same study also identified SPARC (Secreted Protein Acidic and Rich in Cysteine or osteonectin) in colorectal metastases to liver whereas we have detected SPARC mostly in primary colorectal tumors and only in the metastasis of one patient; our results agree concerning the absence of SPARC in normal liver [21]. Periostin has also been reported to be associated with colon cancer metastasis [22,23]; we detected this protein in all the tissue samples studied – it was not specific to malignant tissue but that does not rule out a role in malignant progression. Of note, SPP1, SPARC and periostin have all been implicated in other types of metastatic cancer and are often invoked as possible biomarkers of aggressive tumor types [24-27].

In addition to these concordances with clinical gene expression data, our proteomics-based discovery pipeline identified other proteins that are potential serological biomarkers for colorectal cancer patients: a recent study by Yao and colleagues [28] identified EFEMP2 (EGF-containing fibulin-like extracellular matrix protein 2 or Fibulin 4) and thrombospondin 2 (THBS2) as a potential biomarkers detected in the serum of colorectal cancer patients. In accord with these data, we detected both EFEMP2 and THBS2 in both primary and secondary colon tumors but not in normal tissues. Another example is Tissue Inhibitor of Metalloproteinase-1 (TIMP1), detected in both primary metastatic tumors and metastases to liver in our study, and previously found to be elevated in the serum of colorectal cancer patients and proposed to be not only a good diagnostic factor but also a good predictor and indicator of response to chemotherapy [29,30]. Both EFEMP2 and TIMP1 are also proposed to be of superior value than carcinoembryonic-antigen (CEA), the only biomarker currently used to diagnose and monitor the treatment of colorectal cancer patients [31].

## Conclusions

We demonstrate in this study that we can characterize in detail the composition of the extracellular matrix of normal tissues and tumors using small samples from human colorectal cancer patients. We show that our proteomics approach can robustly define the matrisomes of tumors and matched normal samples. Based on this analysis, we established ECM signatures characteristic of primary metastatic colorectal tumors and their metastases to liver. Further work is needed to determine whether the proteins identified in our study play any functional roles in colorectal tumor progression and metastasis. Future work will aim to characterize the ECM composition of poorly metastatic tumors and of tumors and metastases that do or do not respond to chemotherapy.Although we focused here on proteins common to the three patients studied, it is worth noting that we also identified patient-specific sets of ECM proteins (white areas of the Venn diagrams presented in Figure 2). We hypothesize that these may correlate with some property of the tumors (particular region sampled, stage, etc.) that is beyond the scope of this study to determine due to the small number of tumors examined. Nonetheless, one can postulate that within the signatures defined here are sets of ECM proteins that could serve as novel biomarkers of metastatic potential in primary tumor biopsies and could, furthermore, be used to detect small disseminated metastases that remain undetected by current imaging methods. In order to test these postulates, one needs screening of larger cohorts of patients for presence or absence of these ECM proteins. Our initial comparisons between the ECM protein signatures and mRNA expression datasets support the correlation with metastatic progression of some of the proteins defined – others may well correlate when examined at the protein level (e.g., by immunohistochemistry of tumor tissue microarrays or by serological measurements). ECM proteins are particularly favorable candidate biomarkers since they are abundant, are laid down in characteristic patterns and are readily accessible. ECM protein levels may indeed be more appropriate indicators of tumor properties than mRNA levels since proteins are the operative molecules in the tumor microenvironment and changes occur at that level that are not reflected at the mRNA level because of post-transcriptional processes (translation, stability etc.).

Methods developed by others have already been used effectively to target tumor vascular-specific ECM proteins (splice variants of fibronectin or tenascin) for use in imaging tumors and metastases in mouse models and patients and also for targeting isotopes, drugs and cytokines to tumors for therapeutic applications [32]. A recent study demonstrated that administration of interleukin 12 coupled to an antibody directed against a tumor-specific spliced isoform of fibronectin (a major ECM protein) led to the regression of various tumors including subcutaneous CT26 colon carcinoma tumors [33]. Such approaches could provide sorely needed new strategies for the treatment and management of metastatic colon cancers and we hope that the definition of larger numbers of ECM biomarkers will contribute to improvements in colon cancer patients’ diagnosis, prognosis, treatment and survival.

### Availability of supporting data

In addition to the supporting data included as additional files, the raw GSEA data may be downloaded from: http://rowley.mit.edu/Hynes/Naba_GSEA_ColonCancer/. The raw mass spectrometry data accompanying this publication have been deposited in the public proteomics repository MassIVE (http://massive.ucsd.edu) using the identifier: MSV000078555. The data should be accessible at ftp://MSV000078555:a@massive.ucsd.edu.

## Abbreviations

ECM: Extracellular matrix; LC-MS/MS: Liquid chromatography and tandem mass spectrometry; OGE: Off-gel electrophoresis.w.

## Competing interests

The authors declare that they have no competing interests.

## Authors’ contributions

Conception and design of the experiments: AN, KRC, SAC, KKT, ROH. Development of proteomics methodology: AN, KRC. Acquisition of data: AN, KRC. Analysis and interpretation of data: AN, KRC, CAW, ROH. Writing of the manuscript: AN, KRC, CAW, SAC, KKT, ROH. Study supervision: ROH. All authors have read and approved the submitted manuscript.

## Pre-publication history

The pre-publication history for this paper can be accessed here:

http://www.biomedcentral.com/1471-2407/14/518/prepub

## Supplementary Material

Additional file 1 Supplementary Methods related to the Proteomic analysis of ECM-enriched samples. Click here for file

Additional file 2 **A. Complete proteomics data set.** Proteins are sorted by matrisome divisions (i.e. core matrisome, matrisome associated or other, column A), matrisome categories (column B) and overall confidence score (column BS). B. Subset of extracellular matrix proteins detected in any samples. Data were extracted from Additional file 2A. C. Abundance of ECM proteins characteristic of metastatic colon tumors, metastases or metastatic colon tumors and metastases (see Figure 2E). Numbers represent the abundance of each protein and correspond to the sum of the abundance across the independent samples for each protein (calculated from Additional file 2B, columns F, N and R for normal colon samples; columns V, AD and AL for colon tumor samples; from columns AT, BB and BF for colon tumor metastasis to liver samples and columns BJ and BN for normal liver samples). Dash (-) indicates that the protein was detected in none of the independent samples. White cells are indicative of proteins that were detected in one patient only and thus do not qualify to be part of the matrisome of a given tissue. Click here for file

Additional file 3 **Detailed list of all of the confidently identified peptide spectrum matches (PSMs) from the LC-MS/MS runs of each of the normal tissues and tumor samples analyzed.** Due to its large size, the excel file has been deposited in the public proteomics repository MassIVE and is accessible in the Results directory at ftp://MSV000078555@massive.ucsd.edu/. Click here for file

Additional file 4 **Intra-patient reproducibility (Patient 2).** Venn diagrams represent the intra-patient reproducibility assessed by comparing the ECM composition of two distinct pieces of the same colon tumor (A) or metastasis (B) from patient 2. Click here for file

Additional file 5 **A. List of publicly available clinical gene expression datasets used for GSEA.** B. Values indicate for each gene of the signatures its rank metric scores (used to build Figure 4C and D). Click here for file

Additional file 6 **GSEA Enrichment Plots.** Enrichment plots generated by comparing the metastasis ECM gene set (left panels) and the primary metastatic colon cancer gene set (right panels) defined in this study with four publicly available clinical gene expression data sets (see Additional file 5). Click here for file
