# Supplementary material for: Extracellular matrix signatures of human primary metastatic colon cancers and their metastases to liver
Source: BMC Cancer. 2014 Jul 18;14:518. doi: 10.1186/1471-2407-14-518 (PMC4223627; doi:10.1186/1471-2407-14-518)
Supplement: Additional file 1 — Supplementary Methods related to the Proteomic analysis of ECM-enriched samples. [file 1471-2407-14-518-S1.pdf]

Additional File 1. Supplementary Methods: Proteomic analysis of ECM-enriched samples

Mass Spectrometry

Each off-gel electrophoresis fraction was analyzed with an automated nano LC-MS/MS system, consisting of an Agilent 1100 nano-LC system (Agilent Technologies, Wilmington, DE) coupled to an LTQ Orbitrap XL Fourier transform mass spectrometer (Thermo Fisher Scientific, San Jose, CA) equipped with a nanoflow ionization source (James A. Hill Instrument Services, Arlington, MA). Peptides were eluted from a 10 cm column (Picofrit 75 um ID, New Objectives) packed in-house with ReproSil-Pur C18-AQ 3 um reversed-phase resin (Dr. Maisch, Ammerbuch Germany) using a 120 min. gradient at a flow rate of 200 nl/min to yield ~20 s peak widths. Solvent A was 0.1% formic acid and solvent B was 90% acetonitrile/0.1% formic acid. The elution portion of the LC gradient was 3-6% solvent B in 2 min, 6-31% B in 75 min, 31-60% B in 13 min, 60-90% B in 1 min, and held at 90% B for 5 min. Data-dependent LC-MS/MS spectra were acquired in ~3 s cycles; each cycle was of the following form: one full Orbitrap MS scan at 60,000 resolution followed by 8 MS/MS scans in the ion trap on the most abundant precursor ions using an isolation width of 3 m/z. Dynamic exclusion was enabled with a mass width of +/- 20 ppm, a repeat count of 1 and an exclusion duration of 50 sec. Charge-state screening was enabled along with monoisotopic precursor selection and non-peptide monoisotopic recognition to prevent triggering of MS/MS on precursor ions with unassigned charge or a charge state of 1. The MS/MS spectra were collected using a decision-tree strategy to employ either collision-induced dissociation (CID) or electron-transfer dissociation (ETD) depending on the precursor charge and m/z. ETD spectra were collected if the precursor charge and m/z were: 3 and >650, 4 and >900, or 5 and >950. Otherwise CID spectra were collected. For ETD, fluoranthene was used as the ETD reagent with an anion (automatic gain control) target of 1e5 or 2e5 ions, supplemental activation was not enabled, and the reaction time was dependent on the precursor charge state (precursor charge state - reaction time in msec: +2-100, +3-66.7, +4-50, +5-40, +6-33.3, etc). For CID the normalized collision energy was set to 30 with an activation Q of 0.25 and activation time of 30 msec. All MS/MS spectra were collected with an AGC target ion setting of 1e4 ions.

Protein identification and quantitation

All MS data were interpreted using the Spectrum Mill software package v4.1 beta (Agilent Technologies, Santa Clara, CA). Similar MS/MS spectra acquired on the same precursor m/z

within +/- 60 sec were merged, MS/MS spectra with precursor charge >4 and poor quality MS/MS spectra, which failed the quality filter by not having a sequence tag length > 0 (i.e., minimum of two masses separated by the in-chain mass of an amino acid) were excluded from searching. MS/MS spectra were searched against a UniProt database containing human (78,369 entries) sequences (including isoforms and excluding fragments) downloaded from the UniProt web site on June 30, 2010 with a set of common laboratory contaminant proteins (73 entries) appended. Search parameters included: ESI linear ion-trap scoring parameters for CID spectra, ESI linear ion-trap ETD scoring parameters for ETD spectra, trypsin enzyme specificity (cleavage at KP or RP allowed) with a maximum of four missed cleavages, 35% minimum matched peak intensity, +/- 20 ppm precursor mass tolerance, +/-0.7 Da product mass tolerance, and carbamidomethylation of cysteines and possible carbamylation of N-termini as fixed/mix modifications. Allowed variable modifications were oxidized methionine, deamidation of asparagine, pyro-glutamic acid modification at N-terminal glutamine, pyro-carbamidomethyl modification at N-terminal cysteine, and hydroxylation of proline with a precursor MH+ shift range of -18 to 97 Da. Hydroxyproline was only observed in the proteins known to have it (collagens and proteins containing collagen domains, emilins, etc.) and only within the expected GXPG sequence motifs.

Supplementary Table 2 containing the detailed peptide spectral matches might have some examples not in the expected motif when there is either a proline near the motif for which the spectrum could have had insufficient fragmentation to confidently localize the mass change to a particular residue, or a nearby methionine in the peptide and the spectrum had insufficient fragmentation to localize the mass change to oxidized Met or hydroxyproline. When the motif nX[ST] occurs in a peptide in Supplementary Table 2, this is likely to indicate a site where N-linked glycosylation was removed by the PNGaseF treatment of the sample. While a lowercase n indicates a gene-encoded asparagine residue detected in aspartic acid form, possible mechanisms of modification such as acid-catalyzed deamidation during sample processing versus enzymatic conversion during deglycosylation cannot be explicitly distinguished. Identities interpreted for individual spectra were automatically designated as confidently assigned using the Spectrum Mill autovalidation module to apply target-decoy-based false-discovery rate (FDR) scoring threshold criteria via a two-step auto threshold strategy at the spectral and protein levels. First, peptide mode was set to allow automatic variable range precursor mass filtering with score thresholds optimized to yield a spectral level FDR of 1.6% for each dissociation method and

each precursor charge state in each LC-MS/MS run. Second, protein mode was applied to further filter all the peptide-level validated spectra combined from all LC-MS/MS runs derived from a single tumor using a minimum protein score of 20 and a maximum protein-level FDR of zero. The protein level step filters the results so that each identified protein is comprised of multiple peptides unless a single excellent scoring peptide was the sole match. The above criteria yielded false discovery rates for each sample of <1.0% at the peptide-spectrum match level and <1.4 % at the distinct peptide level as estimated by target-decoy-based searches using reversed sequences. In calculating scores at the protein level and reporting the identified proteins, redundancy is addressed in the following manner: the protein score is the sum of the scores of distinct peptides. A distinct peptide is the single highest scoring instance of a peptide detected through an MS/MS spectrum. MS/MS spectra for a particular peptide may have been recorded multiple times, (i.e. as different precursor charge states, isolated from adjacent OGE fractions, modified by deamidation at Asn or oxidation of Met) but are still counted as a single distinct peptide. When a peptide sequence >8 residues long is contained in multiple protein entries in the sequence database, the proteins are grouped together and the highest scoring one and its accession number are reported.

Relative abundances of proteins were determined using extracted ion chromatograms (XIC's) for each peptide precursor ion in the intervening high resolution FT-MS scans of the LC-MS/MS runs. An individual protein's abundance was calculated as the sum of the ion current measured for all quantifiable peptide precursor ions with MS/MS spectra confidently assigned to that protein. Proteins were considered quantifiable if they were represented in at least two out of the three patients (and in either duplicate samples when available) for normal colon, colon tumor and metastasis and in both normal liver pools of samples. The peak area for the XIC of each precursor ion subjected to MS/MS was calculated automatically by the Spectrum Mill software in the intervening high-resolution MS1 scans of the LC-MS/MS runs using narrow windows around each individual member of the isotope cluster. Peak widths in both the time and *m*/*z* domains were dynamically determined based on MS scan resolution, precursor charge and *m*/*z,* subject to quality metrics on the relative distribution of the peaks in the isotope cluster vs theoretical. Although the determined protein ratios are generally reliable to within a factor of 2-fold of the actual ratio, numerous experimental factors contribute to variability in the determined abundance for a protein. These factors may include incomplete digestion of the protein; widely varying response of individual peptides due to inherent variability in ionization efficiency as well

as interference/suppression by other components eluting at the same time as the peptide of interest, differences in instrument sensitivity over the mass range analyzed, and inadequate sampling of the chromatographic peak between MS/MS scans.
