# Supplementary material for: Extracellular matrix signatures of human primary metastatic colon cancers and their metastases to liver
Source: BMC Cancer. 2014 Jul 18;14:518. doi: 10.1186/1471-2407-14-518 (PMC4223627; doi:10.1186/1471-2407-14-518)
Supplement: Additional file 3 — Detailed list of all of the confidently identified peptide spectrum matches (PSMs) from the LC-MS/MS runs of each of the normal tissues and tumor samples analyzed. Due to its large size, the excel file has been deposited in the public proteomics repository MassIVE and is accessible in the Results directory at ftp://MSV000078555@massive.ucsd.edu/. [file 1471-2407-14-518-S3.docx]

**Additional file 3: Detailed list of all of the confidently identified**

**peptide spectrum matches (PSMs) from the LC-MS/MS runs of each**

**of the normal tissues and tumor samples analyzed**.

Due to its large size, the excel file has been deposited in the public proteomics

repository MassIVE and is accessible in the Results directory at ftp://

MSV000078555@massive.ucsd.edu/.
