# Supplementary figures and images for: Extracellular matrix signatures of human primary metastatic colon cancers and their metastases to liver
Source: BMC Cancer. 2014 Jul 18;14:518. doi: 10.1186/1471-2407-14-518 (PMC4223627; doi:10.1186/1471-2407-14-518)

Additional File 4: Intra-patient reproducibility – Patient 2

Colon Tumor

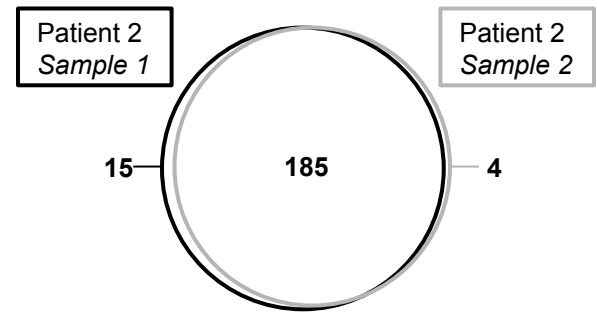

Liver Metastasis

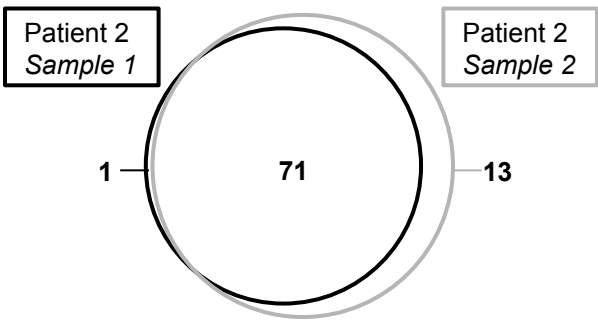

Supplement: Additional file 4 — Intra-patient reproducibility (Patient 2). Venn diagrams represent the intra-patient reproducibility assessed by comparing the ECM composition of two distinct pieces of the same colon tumor (A) or metastasis (B) from patient 2. [file 1471-2407-14-518-S4.pdf]
