# Supplementary material for: Extracellular matrix signatures of human primary metastatic colon cancers and their metastases to liver
Source: BMC Cancer. 2014 Jul 18;14:518. doi: 10.1186/1471-2407-14-518 (PMC4223627; doi:10.1186/1471-2407-14-518)

Additional File 6: GSEA Enrichment Plots

Signature for Colon Tumor Metastasis to Liver

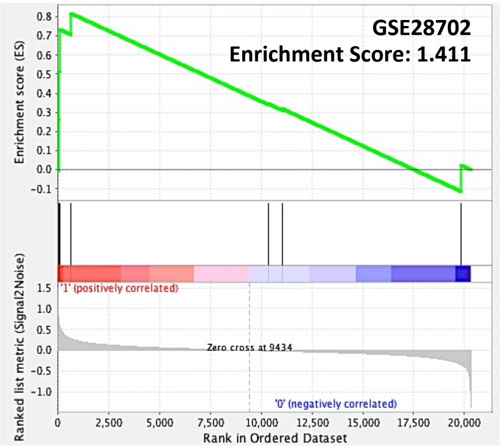

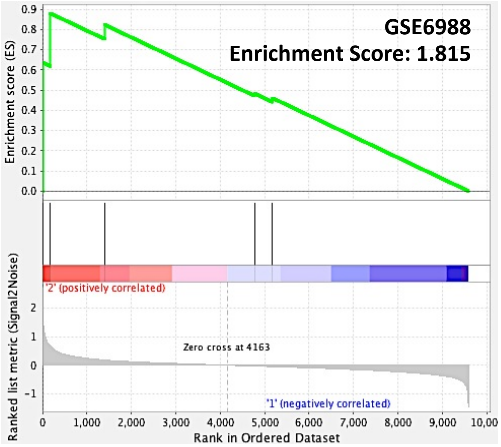

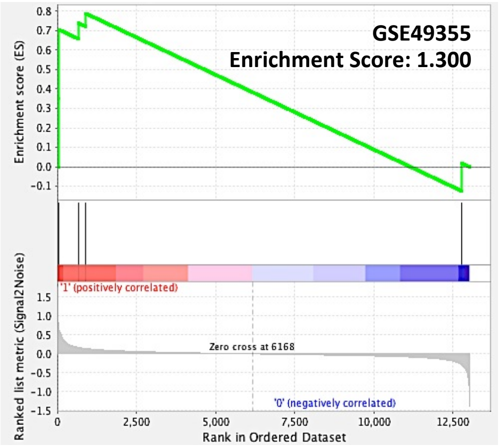

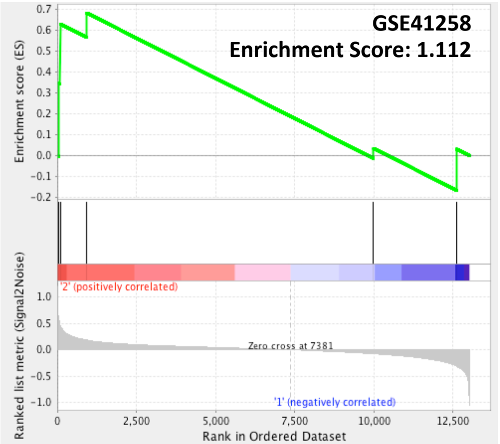

Signature for Primary Colon Tumor

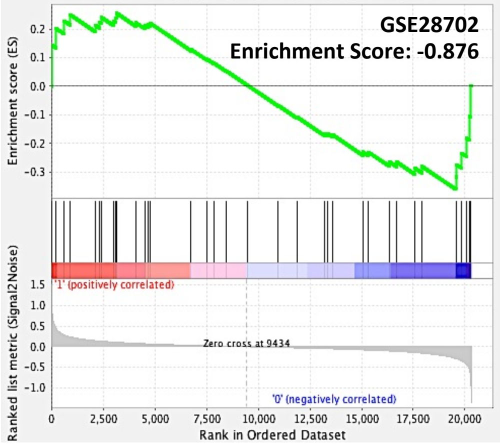

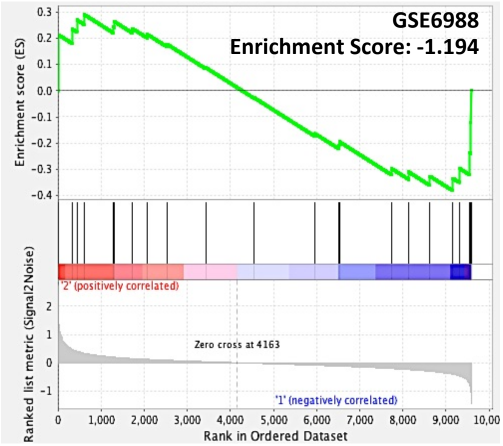

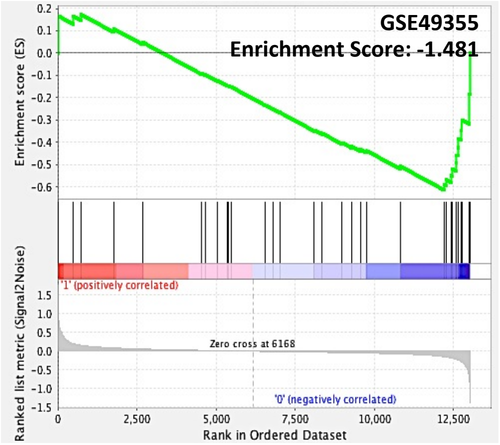

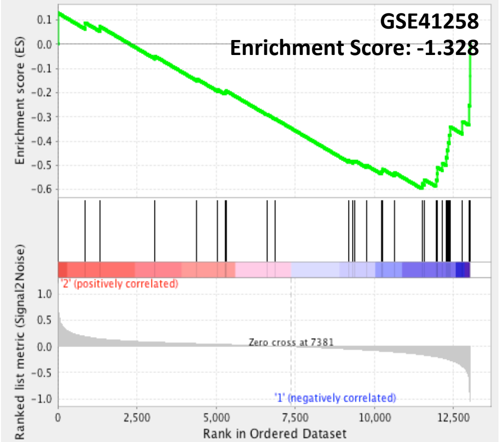

Supplement: Additional file 6 — GSEA Enrichment Plots. Enrichment plots generated by comparing the metastasis ECM gene set (left panels) and the primary metastatic colon cancer gene set (right panels) defined in this study with four publicly available clinical gene expression data sets (see Additional file 5). [file 1471-2407-14-518-S6.pdf]
